# Maximizing transcription of nucleic acids with efficient T7 promoters

**DOI:** 10.1038/s42003-020-01167-x

**Published:** 2020-08-14

**Authors:** Thomas Conrad, Izabela Plumbom, Maria Alcobendas, Ramon Vidal, Sascha Sauer

**Affiliations:** Laboratory for Functional Genomics, Nutrigenomics and Systems Biology, Scientific Genomics Platforms, Max Delbrück Center for Molecular Medicine/Berlin Institute of Health, 13092 Berlin, Germany

**Keywords:** Expression systems, Assay systems, Transcription

## Abstract

In vitro transcription using T7 bacteriophage polymerase is widely used in molecular biology. Here, we use 5′RACE-Seq to screen a randomized initially transcribed region of the T7 promoter for cross-talk with transcriptional activity. We reveal that sequences from position +4 to +8 downstream of the transcription start site affect T7 promoter activity over a 5-fold range, and identify promoter variants with significantly enhanced transcriptional output that increase the yield of in vitro transcription reactions across a wide range of template concentrations. We furthermore introduce CEL-Seq^+^ , which uses an optimized T7 promoter to amplify cDNA for single-cell RNA-Sequencing. CEL-Seq+ facilitates scRNA-Seq library preparation, and substantially increases library complexity and the number of expressed genes detected per cell, highlighting a particular value of optimized T7 promoters in bioanalytical applications.

## Abstract (web summary)

Thomas Conrad et al. identify sequences downstream of transcription start sites that affect T7 promoter activity and use this to generate promoter variants with enhanced transcriptional output. They use the optimized promoters to prepare cDNA libraries for single-cell RNA sequencing with increased complexity compared to those prepared with standard T7 promoters.

## Introduction

Efficient amplification of nucleic acids is critical for many procedures in molecular biology^1^. Over the last decades, polymerase chain reaction (PCR) has been the most widely applied approach to efficiently produce DNA. However, exponential amplification can entail amplification biases, particular in case of low input material. Alternatively, linear amplification of RNA by in vitro transcription (IVT) using single subunit viral RNA polymerases has become core to a host of genomics applications^2^, and is used for large-scale production of RNA. For example, recent single-cell RNA-sequencing (scRNA-seq) methods such as CEL-Seq2^3^, or microdroplet-based (inDrop) procedures^4^, rely on IVT using a T7 bacteriophage promoter and optimized reaction conditions for recombinant T7 RNA polymerase. Furthermore, emerging single-cell DNA-sequencing procedures such as Single-cell whole-genome analyses by linear amplification via transposon insertion (LIANTI^5^), chromatin integration labeling sequencing (CHIL-Seq^6^), scDam&T-seq^7^, or the sciL3 method^8^ fundamentally rely on T7-promoter based IVT.

During transcription initiation, T7 polymerase binds the promoter DNA from nucleotide position −17 to −5 with high specificity, while the DNA double strand is melted from position −4 to +3 to prime RNA synthesis from a GTP nucleotide at position +1^9,10^. The growing RNA:DNA hybrid then expands the initiation bubble from position −4 to +7^11^. Beyond addition of the +8 nucleotide to the nascent RNA molecule, the probability for initiation bubble collapse increases and substantial conformational rearrangements within T7 polymerase mark the transition of the complex into processive elongation^12–14^. The sequence determinants that specify polymerase binding to the core promoter are well characterized, and base substitutions between position −17 and +3 can strongly affect transcriptional output^15–17^. However, to what extent sequences in the extended initiation bubble impact on transcription remains unclear.

Here, we used rapid amplification of cDNA 5′ ends coupled with deep sequencing (5′ RACE-Seq) to test if sequences beyond the +3 nucleotide affect the activity of the T7 promoter. We find that sequence motifs between the +4 and +8 nucleotide have a strong impact on transcriptional output over an unexpected fivefold range, and present a comprehensive list of motifs that can serve as a guideline for the optimization of IVT reactions. We furthermore introduce CEL-seq+ (single-cell RNA-Seq by multiplexed linear amplification+), which uses an optimized T7 promoter for amplification of cDNA from single cells. CEL-Seq+ facilitates the preparation of scRNA-Seq libraries and increases the number of UMI counts and detected genes per cell, demonstrating the utility of optimized T7 promoter sequences for bioanalytical applications.

## Results

### The initially transcribed region affects activity of the T7 promoter

**Fig. 1:**
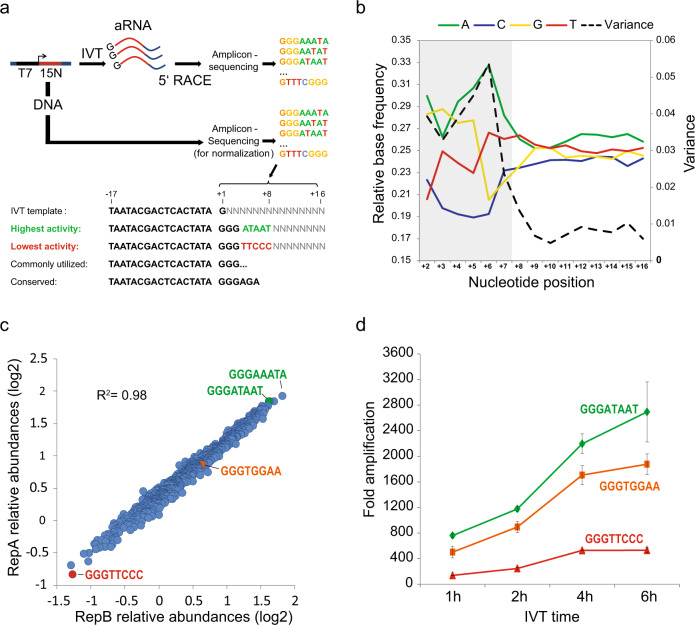
5′ RACE-Seq reveals hyperactive T7 promoter variants. **a** 5^′^RACE-Seq scheme. A 500 bp dsDNA library harboring a T7 promoter template with randomized nucleotide composition from +2 to +16 (highlighted in red) was transcribed in vitro, using T7 RNA polymerase. The resulting 210 nucleotides long RNAs were reverse transcribed, and the 5^′^ end of the respective cDNA was converted into a library for deep sequencing. In parallel, an aliquot of the promoter DNA library was directly sequenced to account for potential sequence bias in the template. **b** Normalized average nucleotide compositions of T7 promoter sequence variants from positions +2 to +16 in amplified RNAs, determined by 5′ RACE-Seq. The region of the extended initiation bubble, which extends from positions −4 to +7, is highlighted in light gray. **c** Differential promoter activity of +4 to +8 sequence motifs determined by 5^′^RACE-seq. Shown are the log2 relative abundances of individual sequence motifs. All promoters contain a G at positions +1 to +3. High correlation was observed between two independent experiments. **d** In vitro transcription reactions comparing +4 to +8 sequence motifs with high, low, and intermediate promoter activity. A 410-nucleotides long RNA was in vitro transcribed for the indicated time points using the displayed promoter variant. Shown is the resulting fold amplification of template DNA. Error bars represent the standard deviation of triplicate experiments.

We used 5′ RACE-Seq to profile the 5′ ends of individual 210 bp long RNAs that were transcribed from 10 ng (~10^10^ copies) of a +2 to +16 randomized T7 promoter template. An aliquot of the promoter library was directly sequenced in parallel to a depth of 2.3 × 10^8^ reads to account for potential sequence biases in the randomized template DNA. We then interrogated the initially transcribed region of >10^8^ sequenced RNA molecules for cross-talk with transcriptional activity (Fig. 1a). After normalization with the template library, base frequencies in transcribed RNA molecules showed high variance from position +2 to +7 and reached baseline levels at nucleotide position +9 (Fig. 1b), indicating substantial sequence preference in the region corresponding to the extended initiation bubble^11^.

**Fig. 2:**
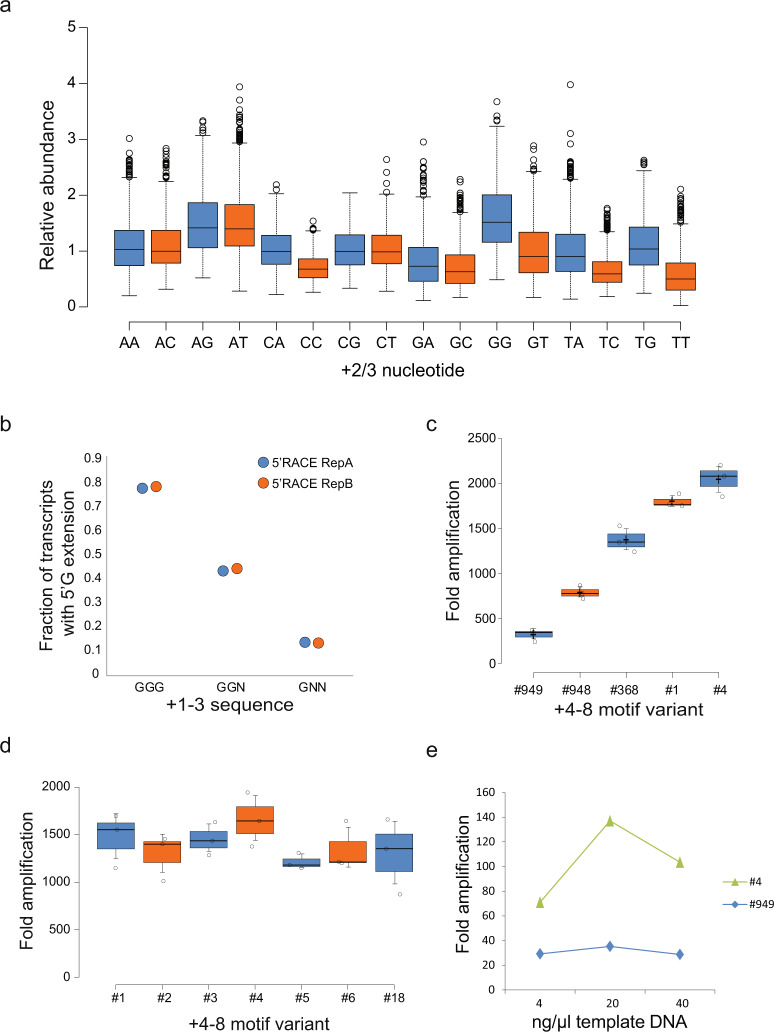
Analysis of T7 promoter sequences. **a** Relative 5′RACE-Seq abundances of T7 promoter +2 to +8 sequence variants, grouped by +2/+3 dinucleotide. Promoters with three guanines at positions +1 to +3 (GGG) showed the highest activity on average (+1G is present in all tested promoter variants). Each whisker plot represents 948–985 +1 to +8 motifs, dependent on homopolymer filtering. Whiskers reach to 1.5× IQR away from the 1st/3rd quartile. **b** Fraction of transcripts with the indicated +1 to +3 sequence, that have an additional G added to the 5′ terminus as a result of polymerase sliding during initiation. Shown are the values from two replicate 5^′^RACE-Seq experiments. **c** Comparison of the IVT activity of +4 to +8 T7 promoter variants with different 5′ RACE-Seq ranks. All T7 promoter variants comprised a GGG sequence at positions +1 to +3 and were used to in vitro transcribe a 410 nucleotides long RNA for 2 h. Shown is the fold amplification relative to the template DNA. Indicated below is the +4 to +8 RACE-Seq rank. **c** The highest ranked +4 to +8 sequence motifs were used for in vitro transcription with T7 polymerase. Shown is the resulting fold amplification of the template DNA after 1 h IVT. **d** The highest ranked +4 to +8 sequence motifs were used for in vitro transcription with T7 polymerase. Shown is the resulting fold amplification of the template DNA after 1 h IVT. **e** Comparison of the IVT activity of two T7 promoter variants using different IVT DNA template concentrations. IVT was performed for 2 h. All error bars represent standard deviation for triplicate experiments.

The obtained sequencing depth provided ~5000-fold oversampling of all possible motifs in the +2 to +8 region, which enabled us to next determine the relative transcriptional activity of individual promoter variants. After normalization with the corresponding motif frequencies in the template library (Supplementary Data 1), transcripts with a G at positions +1 to +3 were transcribed more robustly compared to other +2/3 nucleotide combinations (Fig. 2a), which is in agreement with previous findings and with common guidelines for T7 usage in biomolecular applications. Here, a G triplet may prevent premature dissociation of short abortive transcripts that result from competition between slippage of the nascent RNA and active site translocation. Accordingly, 79% of transcripts that start with a G triplet displayed insertion of an additional G at the 5′ terminus (Fig. 2b). Addition of more than one G was rarely observed (<2% of transcripts with two extra G).

Surprisingly, downstream sequences from +4 to +8 also affected promoter activity over a 5-fold range (Figs. 1c, d and 2c). Importantly, differential transcriptional outputs from individual +4 to +8 promoter variants in 5′ RACE-seq were also recapitulated in individual IVT reactions across the full range of promoter activities (Figs. 1d and 2c, d; Supplementary Data 1). Highly active + 4 to + 8 sequences were generally AT-rich (i.e., AAATA, ATAAT), potentially indicating facilitated DNA double strand melting during initial transcription. However, the observed effects were also sequence-specific, with TTAAA ranking at position #247 in 5′ RACE-Seq, compared to rank #4 for ATAAT (Supplementary Data 1). The distribution of motif activities further suggests extensive combinatorial crosstalk between base positions +4 to +8, which has precluded the detection of transcriptional effects in previous screens based on single-base substitutions^15,16^. As a consequence, the here-reported striking sequence determinant has so far not been taken into account in IVT-based methods. Importantly, initially transcribed sequence motifs still determine transcriptional output in the presence of saturating concentrations of template DNA (Fig. 2e), highlighting their significance for large-scale in vitro synthesis of RNA.

**Fig. 3:**
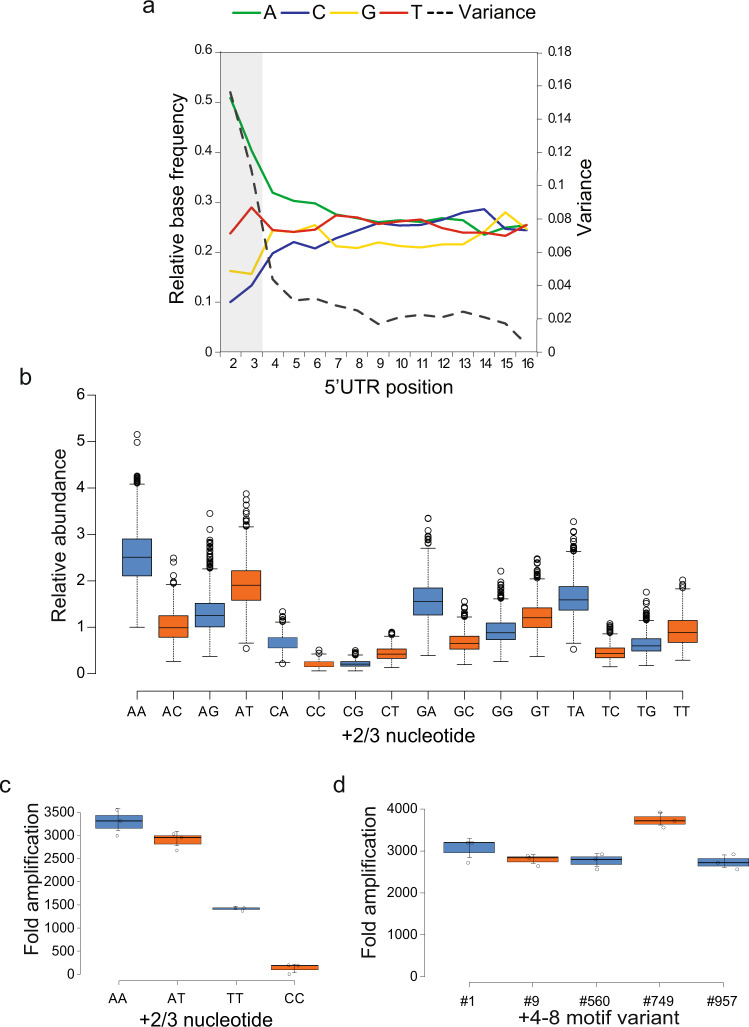
Analysis of SP6 promoter sequences. **a** 5^′^RACE-seq using SP6 polymerase. Normalized average nucleotide composition from position +2 to +16 in RNA transcribed by SP6 RNA polymerase from a randomized SP6 promoter library. Substantial sequence preference was observed until the +3 nucleotide position. **b** Box plot showing relative abundances of +2 to +16 SP6 promoter variants detected in 5^′^RACE-seq, separated by +2/3 dinucleotide sequence. All variants have a G at +1. Promoters with +1 to +3 GAA showed highest activity. Each whisker plot represents 948–985 +1 to +8 motifs, dependent on homopolymer filtering. Whiskers reach to 1.5× IQR away from the 1st/3rd quartile. **c** IVT using high ranking +2 to +8 SP6 promoter variants with the indicated +2/3 dinucleotides. The +2/3 dinucleotide sequence appeared as main determinant of SP6 transcriptional activity. **d** IVT using SP6 promoter templates harboring +1 to +3 GAA followed by +4 to +8 sequence motifs of varying 5^′^RACE-seq rank. IVT was performed for 2 h. Shown is the resulting fold amplification of the template DNA. Sequence elements after +4 showed no effects on SP6 promoter activity. All error bars represent standard deviation for triplicate experiments.

We next tested if initially transcribed sequence determinants were shared by related RNA polymerases. We performed 5′ RACE-seq with RNAs transcribed by SP6 polymerase from a +2 to +16 randomized promoter. In agreement with previous findings^18^, transcriptional output by SP6 polymerase was mostly determined in the +1 to +3 region, with AA and AT being the most actively transcribed +2/3 sequence variants (Fig. 3a–c). In contrast to T7 polymerase, differences between +4 to +8 downstream motifs did not affect transcriptional output by SP6 (Fig. 3d).

### The AT-rich upstream element increases transcription at low template concentrations

**Fig. 4:**
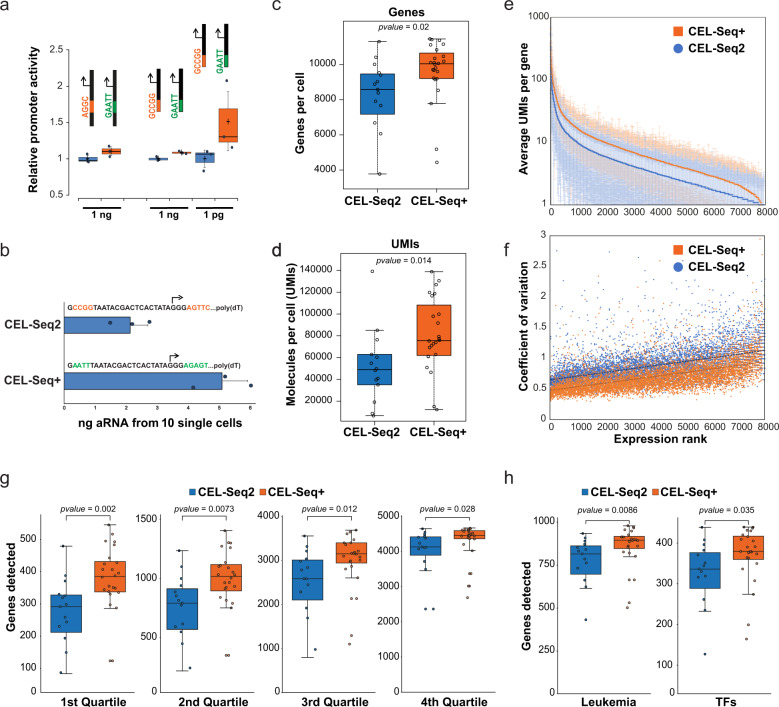
An optimized T7 promoter boosts single-cell RNA-Sequencing. **a** Addition of an AT-rich upstream promoter-flanking region enhances the activity of the T7 promoter at low template concentrations. A 410 nucleotide long RNA was in vitro transcribed for 2 h (1 nanogram template), or 15 h (1 picogram template) in the absence or presence of an AT-rich upstream element as indicated. Shown is the relative RNA yield. The start of the promoter sequence was either located at position 73 (left bars), or at position 6 of the DNA template (middle and right bars). Error bars represent the standard deviation of triplicate experiments. **b** CEL-Seq2 was performed from single K562 cells using the indicated DNA sequences. After reverse transcription and second strand synthesis, cDNA from 10 cells was pooled and in vitro transcribed for 15 h. Purified aRNA was fragmented and quantified on a Tapestation (Agilent). Error bars represent the standard deviation from triplicate experiments. **c** Linear amplification of cDNA in single cells with an optimized T7 promoter (CEL-Seq+) significantly increased the number of detected genes in single-cell RNA-Sequencing (9749 genes per cell on average with new T7 promoter (*n* = 24 cells), 8281 genes per cell on average with conventional T7 promoter (*n* = 14 cells). **d** Linear amplification with an optimized T7 promoter (CEL-Seq+) significantly increased the number of detected molecules in single-cell RNA-Sequencing (85,066 unique molecular identifiers (UMIs)/transcripts per cell on average with new T7 promoter, 53541 UMIs per cell on average with conventional T7 promoter). Statistical analysis was performed using the Mann–Whitney Wilcoxon test. **e** Average UMI count per gene in CEL-Seq2 and CEL-Seq+. Shown are all genes with more than 1 average UMI per cell in both assays (*n* = 7904). Genes are independently sorted by expression rank. Fourteen cells from CEL-Seq+ were randomly chosen for comparison with 14 cells from CEL-Seq2. Error bars show the standard deviation between cells. **f** Coefficient of variation for UMI counts from CEL-Seq2 and CEL-Seq+ for the genes shown in (**e**). **g** Genes from deep sequenced bulk K562 RNA-Seq^20^ were sorted into quartiles by expression level. Shown are the numbers of genes from the indicated bulk quartiles that were detected in individual cells by CEL-Seq2 or CEL-Seq+. **h** Shown are the numbers of genes detected in individual cells that are differentially expressed throughout CML disease progression^20^, or with GO association “DNA binding transcription factor”. Whiskers reach to 1.5× IQR away from the 1st/3rd quartile.

It was previously shown that introduction of a short AT-rich sequence element upstream of the T7 core promoter increases binding affinity of the polymerase for the DNA template^19^. However, the impact on transcription remained unclear. In our hands, introduction of a short AT-rich sequence at position −21 to −18 only led to a modest increase in transcriptional output of 1.1-fold (Fig. 4a). Similar results were obtained when the promoter was moved to the 5′ terminus of the template DNA, emphasizing the downstream promoter flanking region as main modulator of transcriptional activity under normal conditions (Fig. 4a). However, enhanced promoter affinity did translate into a 1.5-fold increase in transcriptional output when the template concentration was reduced to 1 pg/µl, a template amount typically observed in single cell derived cDNA libraries (Fig. 4a).

### Enhanced single cell RNA-Seq with an optimized T7 promoter

A host of single cell genomics methods rely on amplification of scarce nucleic acids via T7-based amplification. To demonstrate the utility of an optimized T7 promoter for these applications, we set out to rationally design an optimized primer for single-cell RNA sequencing based on the popular CEL-Seq2 procedure^3^. Here, mRNA from single cells is captured via hybridization of an oligo d(T) primer fused to an upstream T7 promoter sequence, then reverse transcribed and amplified by IVT with T7 polymerase (Supplementary Fig. 1). The transcribed antisense RNA (aRNA) is again converted into cDNA and processed into a library for deep sequencing. Successful preparation of a sequencing library strongly depends on the efficiency of the T7-based amplification step. Furthermore, a main characteristic of single-cell RNA-seq is a high “drop out” rate, i.e., features that are present in an individual cell but escape detection. Accordingly, scRNA-seq data is inherently shallow, meaning that most expressed genes are only represented by a small number of transcript counts. This is because many mRNA molecules are not initially captured by hybridization, or are subsequently lost in one of the downstream steps of library preparation, all of which are not 100% efficient. We therefore hypothesized that additional copies generated by a more efficient T7 polymerase may increase the probability especially of lowly abundant transcripts to be represented in the final sequencing output, corresponding to enhanced sensitivity of scRNA-seq.

The T7 promoter in the standard CEL-Seq2 primer contains a CG-rich 5 bp upstream region, which was replaced by the AT-rich upstream motif (Fig. 4b). Surprisingly, insertion of the high-ranking +4 to +8 motif #4 into the CEL-Seq2 primer only led to a modest increase in aRNA yield (Supplementary Fig. 2). We speculated that this was due to less efficient mRNA capture by the extended primer. Therefore, in order to maintain the shortest possible primer length and to minimize the overall sequence change, we instead only inserted a GA dinucleotide at T7-position +3/+4 in the CEL-Seq2 primer, which elevated the 5^′^RACE-Seq rank of the +4 to +8 region from #177 to #66 (Supplementary Data 1). Following mRNA capture with the optimized primer, we observed a robust ~2.5-fold increase in aRNA amplification from pooled cDNA of 10 single K562 cells, which facilitated library preparation from scarce material (Fig. 4b). Most importantly, leveraging the new T7 promoter sequence for CEL-seq+ substantially increased the number of detected genes (average 9749 vs. 8281), and the number of unique molecular identifiers (UMIs; average 85066 vs. 53541) from single cells (Fig. 4c). Detection of almost 10,000 genes per cell likely approaches the entire set of expressed genes present in a single cell at any given time. In addition to higher detection rates, expression levels of individual genes were measured with higher accuracy by CEL-Seq+ across the entire gene expression spectrum, as reflected by higher UMI counts and a consistently lower coefficient of variation (CV) (Fig. 4e, f). As an additional benchmark we next divided the genes detected in deep sequenced bulk K562 RNA-Seq^20^ into expression quartiles and tested their recovery in CEL-seq2 vs. CEL-Seq+. Enhanced recovery of genes from lower expression quartiles in CEL-Seq+ suggested that improved linear amplification based on the here introduced T7 promoter sequences directly translates into substantially increased sensitivity of single-cell genomics applications (Fig. 4g). At the same time, the detection rate of transcription factors, chromatin binders, or genes involved in CML disease progression was significantly increased (Fig. 4h; Supplementary Fig. 3). Importantly, 64 transcription factors were exclusively detected by CEL-Seq+, including many with known relevance for leukemia (i.e., TLX^21^), CML tumor stem cells (i.e., PPARG^20^), CML cancer cell metabolism (i.e., SIX1^20^), or response to treatment (i.e., PBX1^20^). Accordingly, single cell studies aiming at a mechanistic understanding of tumor biology may broadly benefit from application of an enhanced T7 promoter in CEL-Seq+. Furthermore, important low-abundant regulatory genes are usually missed by microdroplet-based single-cell RNA-sequencing methods that rather produce particularly shallow transcriptomes. Also microdroplet-based methods such as inDrop^4^ may therefore benefit from using the here reported T7 promoters to increase gene detection rates.

## Discussion

In general, uniform and accurate amplification of nucleic acids is important when starting material is scarce and precious. The optimized T7 promoter sequences presented here can be readily applied in various IVT-based methods to boost linear amplification of nucleic acids. This is of particular interest for single-cell DNA- and RNA-sequencing approaches, and for nucleic acids based diagnostics of scarce clinical material such as circulating tumor cells^22^. In current biomolecular approaches, the initially transcribed region either contains random linker DNA or represents the 5′end of the amplified sequence, resulting in highly variable outcomes. Accordingly, an optimized T7 promoter with reliably high transcriptional output is of particular relevance for T7-based biosensors that need to amplify different target molecules with consistent efficiency. Importantly, the optimized T7 promoters presented here enhance transcription across a wide range of template concentrations, so that any IVT-based application can be readily enhanced by up to 500% by simply following our sequence recommendations. In addition to bioanalytical application, the here introduced promoter sequences may thus help to improve large-scale production of (modified) RNA or protein in vitro.

## Methods

### Generation of 5^′^RACE-seq libraries

Totally, 10 ng of dsDNA template (T7_15N/SP6_15N) containing a T7/SP6 RNA polymerase promoter randomized from position +2 to +16 (gBlocks Gene Fragments, Integrated DNA Technologies) was used as input for IVT in a 20 µl reaction using 1.5 µl T7 or T6 RNA polymerase mix and 7.5 mM each of GTP, ATP, CTP, and UTP from the HiScribe T7/SP6 RNA Synthesis Kit (NEB E2040S, E2070S). After 2 h incubation, RNA was purified with 1.6 volumes RNAClean XP beads (Beckman Coulter), and residual template DNA digested using the TURBO DNA-freeTM Kit (Invitrogen AM1907). RNA yield was assayed using the QubitTM RNA Assay Kit. Next, 500 ng of RNA was reverse transcribed using 6.66 U/µl SuperScript^™^ IV (Invitrogen 18090010) and 1.2 µM RT oligonucleotide. Following RNAseA and RNAseH treatment, cDNA was purified using 1.6 volumes Ampure XP beads and eluted in 10 µl water. A poly(A)-tailing reaction was carried out using 3 U/µl terminal transferase (Invitrogen 10533). Second-strand cDNA synthesis was performed in 50 µl with 0.5 µM Oligo i5_5N_dT20VN, 2000 U Q5^®^ High-Fidelity DNA Polymerase, and 0.2 mM dNTPs, using the temperature cycle 98 °C for 30 s; 55 °C for 30 s; 72 °C for 5 min. Next, 2 µL Exonuclease 1 and 48 µL water were added and the reaction was incubated for 1 h at 37 °C. Then, dsDNA was purified with 1.6 volumes AMPure XP beads, and eluted in 22 µL water. Sequencing adapters were introduced during 8 cycles of PCR with KAPA HiFi HotStart. Libraries were purified twice with 1.2 volumes AMPure XP beads and sequenced using a NextSeq500 instrument from Illumina in paired-end mode (2 × 35), detecting the randomized 15mer sequence in Read1 and a 5 base unique molecular identifier (UMI) in Read2. For background library preparation, a total of 2.5 ng of dsDNA template (T7_15N/SP6_15N) was amplified by 6 cycles of PCR using KAPA HiFi HotStart Polymerase, P5 and BG_T7/BG_SP6 sequencing adapters. Libraries were purified twice with 0.9 volumes AMPure XP beads and sequenced in single end mode using a NextSeq 500 instrument from Illumina. The first 15 nucleotides were used for data analysis.

### 5′RACE-seq sequence data analysis

Raw reads from 5^′^RACE-seq libraries were filtered for PhiX using Bowtie 2 (http://bowtie-bio.sourceforge.net/bowtie2/index.shtml). Duplicate 15mer-UMI combinations were removed. Forward reads harboring 15mer sequences were selected for the presence of a C at position 16, followed by AA, CA or CC at position 17 and 18 (to accommodate for Cs added by the reverse transcriptase to the end of cDNAs, as well as for T7 polymerase sliding in promoter variants with transcription starting on triple G sequences), followed by a polyA sequence. After reverse-complementing the 15mer, 10mers corresponding to position +2 to +11 in the RNA were mapped against the constant sequences of the sequencing library and T7 template DNA using Bowtie 2 allowing one mismatch, to remove spurious contaminating sequences. After filtering, 35 × 10^6^ and 84 × 10^6^ reads were used for motif analysis, respectively. For background libraries, reads were filtered for PhiX using Bowtie 2, resulting in 232 × 10^6^ total reads for motif analysis. To determine relative abundances of +2 to +8 promoter variants, reads with identical +2 to +8 sequence were pooled, and the resulting read counts were divided by the total number of filtered reads and by the corresponding counts in background libraries. Homopolymeric motifs were removed from the analysis because reads containing long stretches of G appeared to be disproportionally removed by the real time analysis software application of the Illumina NextSeq 500 sequencer (base positions corresponding to dark sequencing cycles correspond to G in this two fluorescence channel sequencing system).

### IVT for validations

One ng of dsDNA template (gBlocks Gene Fragments, Integrated DNA Technologies) was in vitro transcribed in a 20 µl reaction using 1.5 µl T7 or T6 RNA polymerase mix and 7.5 mM each of GTP, ATP, CTP, and UTP from the HiScribe T7/SP6 RNA Synthesis Kit (NEB E2040S, E2070S). RNA was purified with 1.6 volumes RNAClean XP beads, followed by elution in 20 µL water. RNA was quantified using the QubitTM RNA Assay Kit. For testing of CEL-seq adapter variants, 1 pg of double stranded 330 bp CEL-seq cDNA mimics were in vitro transcribed using the HiScribe T7 RNA Synthesis Kit. RNA was purified with 1.6 volumes RNAClean XP beads, and eluted in 10 µL water. RNA was quantified using a High Sensitivity RNA ScreenTape (Agilent 5067–5579) on an Agilent Tapestation.

### Single-cell RNA-sequencing

CEL-Seq2 was performed as described in^3^. In brief, single cells were FACS sorted using a BD Aria III device into 96 well plates with barcoded primers (0.4 µM) in lysis buffer (110 µM dNTPs, 0.007% Triton X-100, 1.4 U SUPERaseIn) and stored at −80 °C. After thawing, plates were heated to 72 °C for 3 min and incubated for 10 min at 10 °C. Four microlitre Superscript II reaction mix were added at room temperature and the plate was incubated for 1 h at 42 °C followed by heat inactivation for 10 min at 70 °C. Second strand synthesis was performed at 16 °C for 2 h with the NEBNext^®^ Ultra II Non-Directional RNA Second Strand Synthesis Module (NEB E6111S). Single cell reactions were pooled separately for CEL-Seq2 or CEL-Seq+ primers, purified with 1.2 volumes of Ampure XP, and in vitro transcribed for 15 h using the MEGAscript T7 kit. After EXOSAP-IT treatment (Thermo, 78201) and fragmentation for 1.5 min (NEB E6150S), RNA was cleaned up by 1.8 volumes of Ampure XP. The fragmented RNA was combined with HEX-primer and dNTPs and incubated at 65 °C for 5 min followed by addition of superscript II mic and incubation at 25 °C for 10 min and 42 °C for 1 h. The cDNA was amplified with 12 cycles of PCR using KAPA HiFi HotStart ReadyMix. Final reactions were cleaned up with 0.8 fold Ampure XP and sequenced on a MiniSeq (Illumina) instrument.

### Single-cell RNA-sequencing data analysis

Fastq files from each dataset were processed by zUMI software (version 2.5) for filtering, single cell demultiplexing and mapping the reads into genes. Filtering was done by using default parameters of zUMI (minimum reads per cell equal to 100), reads were mapped to hg38 genome assembly version and gencode (v27) annotation for gene read count. From each dataset, the output matrix files from zUMI were accessed by Seurat (version 3). Reads from intronic and exonic region of the genes were included and no further coverage filtering was applied.

### Statistics and reproducibility

Standard deviation between triplicate IVT experiments was determined using student’s *t* test. Numbers of detected genes and UMIS in CEL-Seq2 and CEL-Seq+ were compared using the Mann–Whitney Wilcoxon test.

### Reporting summary

Further information on research design is available in the Nature Research Reporting Summary linked to this article.

## Supplementary information

Reporting Summary

Supplementary Information

Description of Additional Supplementary Files

Supplementary Data 1

Supplementary Data 2

## Data Availability

Oligonucleotides used in this study are listed in Supplementary Data 2. Sequencing data was deposited in the ArrayExpress database (http://www.ebi.ac.uk/arrayexpress) and is available under the accession No. E-MTAB-8508.
