# Supplementary material for: Maximizing transcription of nucleic acids with efficient T7 promoters
Source: Commun Biol. 2020 Aug 14;3:439. doi: 10.1038/s42003-020-01167-x (PMC7429497; doi:10.1038/s42003-020-01167-x)
Supplement: Supplementary file 2 — Supplementary Information [file 42003_2020_1167_MOESM2_ESM.pdf]

Supplementary Figure 1

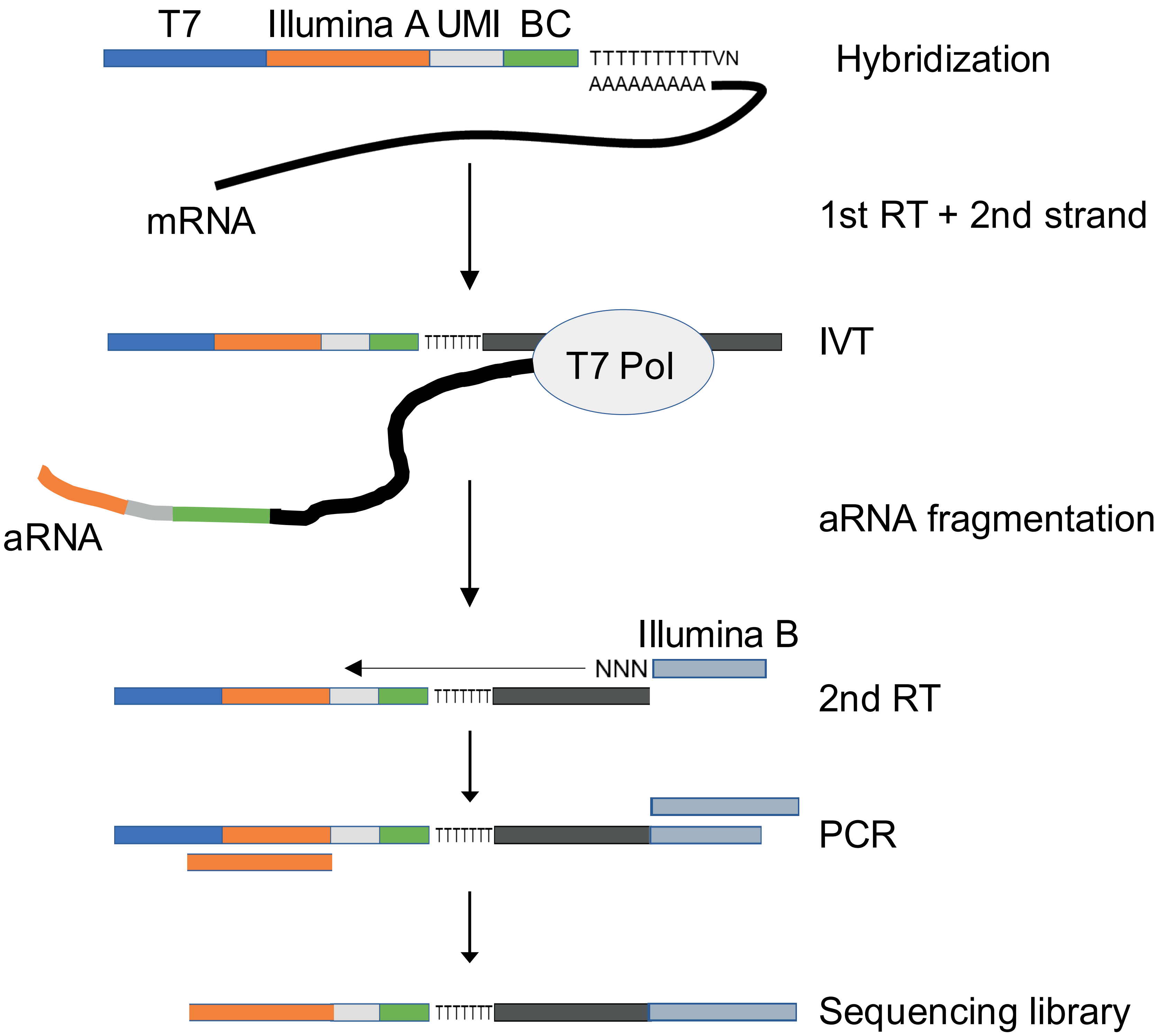

Supplementary Figure 1.
CEL-Seq2 workflow. Shown on top is a schematic of the CEL-Seq2 primer (T7 = T7 promoter; Illumina A = partial sequencing adapter A; UMI = unique molecular identifier; BC = cellular barcode). Polyadenylated RNA from single cells is captured via the oligo d(T) terminus of the CEL-Seq2 primer. After reverse transcription and second strand synthesis, mRNA sequences are amplified by in vitro transcription. Antisense RNA (aRNA) is fragmented, and reverse transcribed using a random hexamer primer fused to partial sequencing adapter B (Illumina B). Sequencing adapters are completed, and the library is amplified in a final PCR step.

Supplementary Figure 2

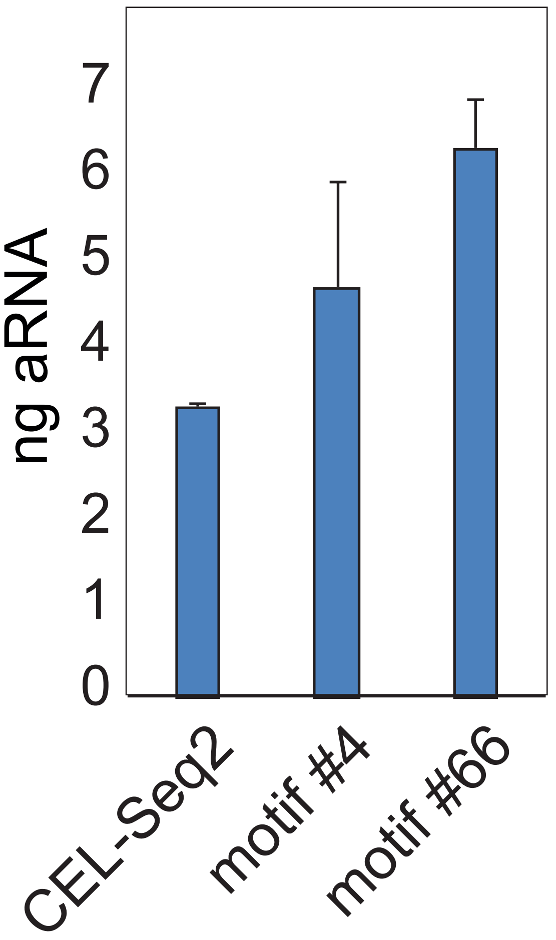

Supplementary Figure 2.
CEL-Seq2 was performed from single K562 cells using the original T7 promoter sequence or the indicated +4 to +8 motifs. After reverse transcription and second strand synthesis, cDNA from 10 cells was pooled and in vitro transcribed for 15 h. Purified RNA was fragmented and quantified on a Tapestation (Agilent). Error bars represent the standard deviation from triplicate experiments.

Supplementary Figure 3

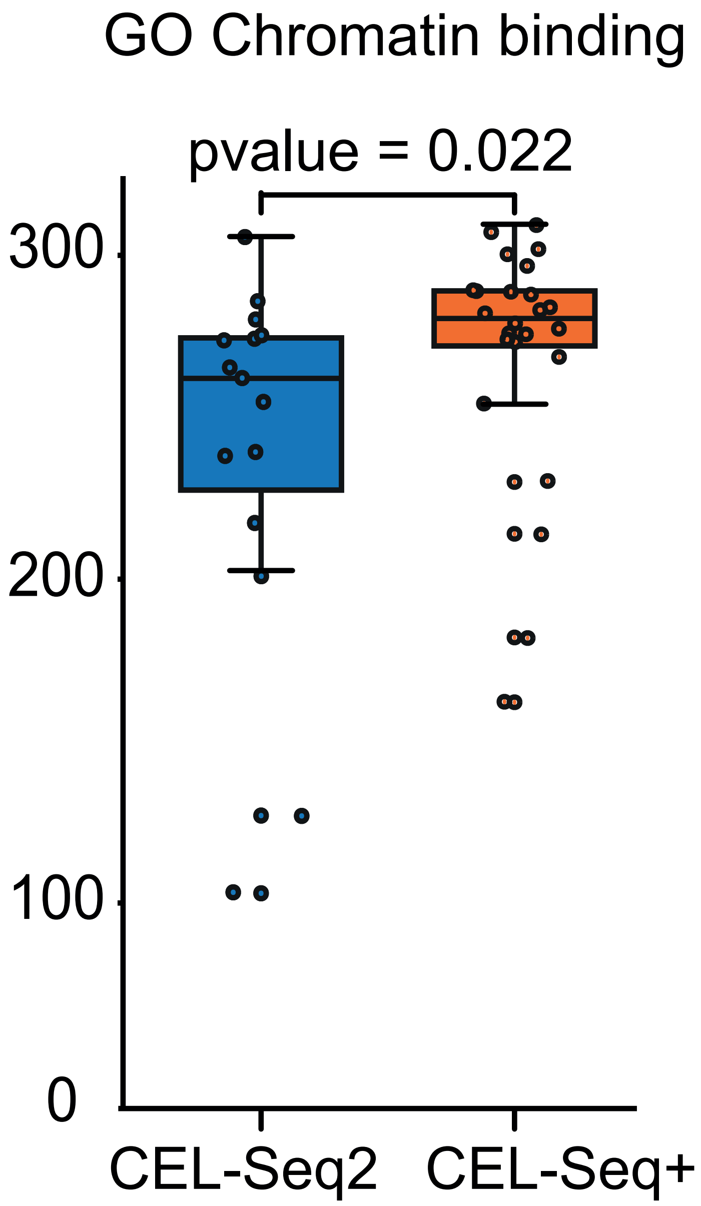

Supplementary Figure 3.
Shown are the numbers of genes detected in individual cells with the GO association “chromatin binding”. Whiskers reach to 1.5x IQR away from the 1st/3rd quartile.
