# Supplementary material for: Maximizing transcription of nucleic acids with efficient T7 promoters
Source: Commun Biol. 2020 Aug 14;3:439. doi: 10.1038/s42003-020-01167-x (PMC7429497; doi:10.1038/s42003-020-01167-x)
Supplement: Supplementary file 3 — Description of Additional Supplementary Files [file 42003_2020_1167_MOESM3_ESM.pdf]

Description of additional supplementary files

Supplementary Data 1: 5'RACE ranked motifs

Supplementary Data 2: DNA oligos used in this study
